# Iodidobis(η^5^-penta­methyl­cyclo­penta­dien­yl)titanium(III)

**DOI:** 10.1107/S1600536810040110

**Published:** 2010-10-13

**Authors:** Monty Kessler, Anke Spannenberg, Uwe Rosenthal

**Affiliations:** a Leibniz-Institut für Katalyse e. V. an der Universität Rostock, Albert-Einstein-Strasse 29a, 18059 Rostock, Germany

## Abstract

In the title complex mol­ecule, [Ti(C_10_H_15_)_2_I], the paramagnetic Ti(III) atom is coordinated by two penta­methyl­cyclo­penta­dienyl (Cp*) ligands and one iodide ligand. The two Cp* ligands are in a staggered orientation. The coordination geometry at the titanium atom can be described as distorted trigonal-planar.

## Related literature

For related bis­(*η* ^5^-penta­methyl­cyclo­penta­dien­yl)titanium(III) halides, Cp*_2_Ti*X*, see: Pattiasina *et al.* (1987 ▶) (*X* = Cl); Herzog *et al.* (1994 ▶) (*X* = F). For the mol­ecular structure of Cp*_2_TiF, see: Lukens *et al.* (1996 ▶). For bis­(*η* ^5^-tetra­methyl­cyclo­penta­dien­yl)titanium(III) halides, see: Troyanov *et al.* (1993 ▶).
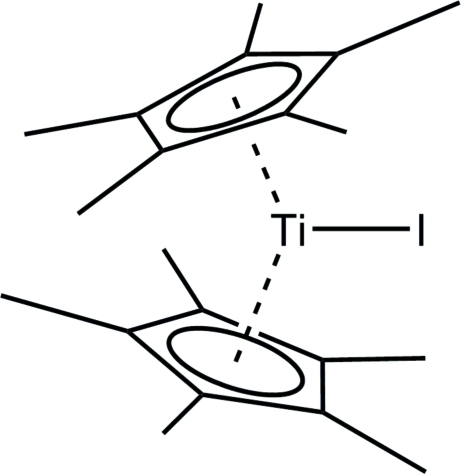


## Experimental

### 

#### Crystal data

[Ti(C_10_H_15_)_2_I]
*M* *_r_* = 445.24
Monoclinic, 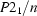

*a* = 8.5513 (3) Å
*b* = 14.1353 (5) Å
*c* = 16.9547 (6) Å
β = 103.158 (3)°
*V* = 1995.60 (12) Å^3^
*Z* = 4
Mo *K*α radiation
μ = 1.97 mm^−1^
*T* = 150 K
0.60 × 0.27 × 0.20 mm

#### Data collection

Stoe IPDS II diffractometer
Absorption correction: numerical (*X-SHAPE* and *X-RED32*; Stoe & Cie, 2005 ▶) *T*_min_ = 0.451, *T*_max_ = 0.875
23992 measured reflections
5395 independent reflections
3921 reflections with *I* > 2σ(*I*)
*R*_int_ = 0.036

#### Refinement

*R*[*F* ^2^ > 2σ(*F* ^2^)] = 0.025
*wR*(*F* ^2^) = 0.052
*S* = 0.86
5395 reflections
209 parameters
H-atom parameters constrained
Δρ_max_ = 0.65 e Å^−3^
Δρ_min_ = −0.43 e Å^−3^

### 

Data collection: *X-AREA* (Stoe & Cie, 2005 ▶); cell refinement: *X-AREA*; data reduction: *X-AREA*; program(s) used to solve structure: *SHELXS97* (Sheldrick, 2008 ▶); program(s) used to refine structure: *SHELXL97* (Sheldrick, 2008 ▶); molecular graphics: *XP* in *SHELXTL* (Sheldrick, 2008 ▶); software used to prepare material for publication: *SHELXL97*.

## Supplementary Material

Crystal structure: contains datablocks I, global. DOI: 10.1107/S1600536810040110/is2610sup1.cif

Structure factors: contains datablocks I. DOI: 10.1107/S1600536810040110/is2610Isup2.hkl

Additional supplementary materials:  crystallographic information; 3D view; checkCIF report

## Figures and Tables

**Fig. 1.**
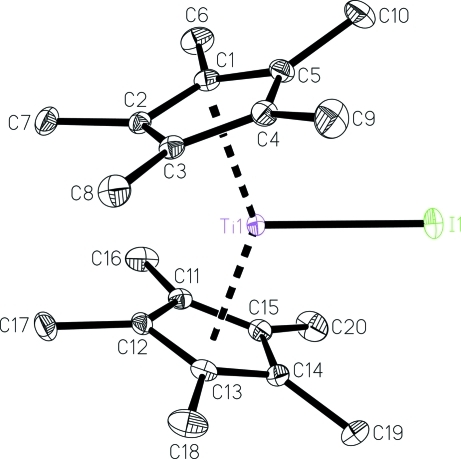
The molecular structure of the title compound showing the atom-labelling scheme. Hydrogen atoms are omitted for clarity. Thermal ellipsoids are drawn at the 30% probability level.

## supplementary crystallographic information

### Comment

The title compound, Cp*_2_TiI, was first synthesized by Pattiasina *et al.* (1987) *via* salt metathesis of Cp*_2_TiCl. Using Cp*_2_TiF as starting material we obtained Cp*_2_TiI in high yields and single crystals by recrystallization from *n*-pentane at -78 °C. The X-ray crystal structure analysis of Cp*_2_TiI confirms its monomeric structure probably due to the steric demand of the pentamethylcyclopentadienyl ligands. The trivalent paramagnetic titanium center is coordinated by one iodide ligand and by two Cp*-ligands in a *η*^5^-coordination mode. The Cp*-ligands are in a staggered orientation. The coordination geometry at the titanium is distorted trigonal planar (CE1—Ti1—CE2 = 142.4, CE1—Ti1—I1 = 109.2 and CE2—Ti1—I1 = 108.4°; CE1 = centroid of C1—C5 and CE2 = centroid of C11—C15). A similar distortion is observed for the known bis(*η*^5^-pentamethylcyclopentadienyl)-titanium(III) halides Cp*_2_TiF (Herzog *et al.*, 1994) and Cp*_2_TiCl (Pattiasina *et al.*, 1987). The Ti1—I1 bond length of 2.7508 (3) Å is close to that in the related complex (*η*^5^-C_5_HMe_4_)_2_TiI [Ti1—I1 = 2.759 (2) Å] (Troyanov *et al.*, 1993).

### Experimental

A mixture of Cp*_2_TiF (0.350 g, 1.04 mmol) and LiI (0.223 g, 1.67 mmol) was suspended in 25 ml diethyl ether and stirred at room temperature overnight. The solvent was removed *in vacuo* and the dark green residue was extracted with *n*-hexane. The filtrate was concentrated to dryness *in vacuo* and the solid residue was recrystallized from *n*-pentane to give dark blue-green needles. Yield: 0.431 g (0.97 mmol, 93%).

### Refinement

All H atoms were placed in idealized positions with d(C—H) = 0.98 and refined using a riding model, with *U*_iso_(H) fixed at 1.5*U*_eq_(C).

### Crystal data

**Table:**

[Ti(C_10_H_15_)_2_I]	*F*(000) = 900
*M**_r_* = 445.24	*D*_x_ = 1.482 Mg m^−^^3^
Monoclinic, *P*2_1_/*n*	Mo *K*α radiation, λ = 0.71073 Å
Hall symbol: -P 2yn	Cell parameters from 5078 reflections
*a* = 8.5513 (3) Å	θ = 1.8–29.6°
*b* = 14.1353 (5) Å	µ = 1.97 mm^−^^1^
*c* = 16.9547 (6) Å	*T* = 150 K
β = 103.158 (3)°	Needle, dark blue-green
*V* = 1995.60 (12) Å^3^	0.60 × 0.27 × 0.20 mm
*Z* = 4	

### Data collection

**Table:**

Stoe IPDS II diffractometer	5395 independent reflections
Radiation source: fine-focus sealed tube	3921 reflections with *I* > 2σ(*I*)
graphite	*R*_int_ = 0.036
ω scans	θ_max_ = 29.2°, θ_min_ = 1.9°
Absorption correction: numerical (*X-SHAPE* and *X-RED32*; Stoe & Cie, 2005)	*h* = −11→11
*T*_min_ = 0.451, *T*_max_ = 0.875	*k* = −19→19
23992 measured reflections	*l* = −22→23

### Refinement

**Table:**

Refinement on *F*^2^	Primary atom site location: structure-invariant direct methods
Least-squares matrix: full	Secondary atom site location: difference Fourier map
*R*[*F*^2^ > 2σ(*F*^2^)] = 0.025	Hydrogen site location: inferred from neighbouring sites
*wR*(*F*^2^) = 0.052	H-atom parameters constrained
*S* = 0.86	*w* = 1/[σ^2^(*F*_o_^2^) + (0.0282*P*)^2^] where *P* = (*F*_o_^2^ + 2*F*_c_^2^)/3
5395 reflections	(Δ/σ)_max_ = 0.001
209 parameters	Δρ_max_ = 0.65 e Å^−^^3^
0 restraints	Δρ_min_ = −0.43 e Å^−^^3^

### Special details

**Table:**

Geometry. All e.s.d.'s (except the e.s.d. in the dihedral angle between two l.s. planes) are estimated using the full covariance matrix. The cell e.s.d.'s are taken into account individually in the estimation of e.s.d.'s in distances, angles and torsion angles; correlations between e.s.d.'s in cell parameters are only used when they are defined by crystal symmetry. An approximate (isotropic) treatment of cell e.s.d.'s is used for estimating e.s.d.'s involving l.s. planes.
Refinement. Refinement of *F*^2^ against ALL reflections. The weighted *R*-factor *wR* and goodness of fit *S* are based on *F*^2^, conventional *R*-factors *R* are based on *F*, with *F* set to zero for negative *F*^2^. The threshold expression of *F*^2^ > σ(*F*^2^) is used only for calculating *R*-factors(gt) *etc*. and is not relevant to the choice of reflections for refinement. *R*-factors based on *F*^2^ are statistically about twice as large as those based on *F*, and *R*- factors based on ALL data will be even larger.

### Fractional atomic coordinates and isotropic or equivalent isotropic displacement parameters (Å^2^)

**Table:**

	*x*	*y*	*z*	*U*_iso_*/*U*_eq_	
C1	0.1702 (2)	0.69392 (15)	0.10016 (11)	0.0231 (4)	
C2	0.1239 (2)	0.78575 (13)	0.07150 (11)	0.0200 (4)	
C3	0.2412 (2)	0.82110 (13)	0.03163 (11)	0.0207 (4)	
C4	0.3572 (2)	0.74932 (15)	0.03365 (12)	0.0239 (4)	
C5	0.3119 (2)	0.67040 (14)	0.07461 (13)	0.0253 (4)	
C6	0.0717 (3)	0.62684 (17)	0.13752 (15)	0.0376 (5)	
H6A	0.0090	0.6627	0.1691	0.056*	
H6B	0.1430	0.5826	0.1732	0.056*	
H6C	−0.0012	0.5914	0.0947	0.056*	
C7	−0.0419 (2)	0.82456 (16)	0.06611 (14)	0.0310 (5)	
H7A	−0.1202	0.7868	0.0276	0.047*	
H7B	−0.0464	0.8904	0.0476	0.047*	
H7C	−0.0673	0.8218	0.1196	0.047*	
C8	0.2339 (3)	0.91097 (16)	−0.01645 (13)	0.0309 (5)	
H8A	0.3413	0.9391	−0.0071	0.046*	
H8B	0.1601	0.9555	0.0006	0.046*	
H8C	0.1954	0.8969	−0.0742	0.046*	
C9	0.4955 (3)	0.7556 (2)	−0.00683 (16)	0.0401 (6)	
H9A	0.4566	0.7465	−0.0653	0.060*	
H9B	0.5745	0.7065	0.0149	0.060*	
H9C	0.5460	0.8180	0.0035	0.060*	
C10	0.3827 (3)	0.57250 (16)	0.07894 (18)	0.0431 (6)	
H10A	0.3171	0.5328	0.0367	0.065*	
H10B	0.3848	0.5449	0.1322	0.065*	
H10C	0.4924	0.5761	0.0708	0.065*	
C11	0.2539 (2)	0.87291 (14)	0.26881 (12)	0.0220 (4)	
C12	0.2530 (2)	0.94111 (14)	0.20774 (12)	0.0218 (4)	
C13	0.4142 (2)	0.96209 (13)	0.20569 (12)	0.0223 (4)	
C14	0.5137 (2)	0.91117 (14)	0.26920 (13)	0.0246 (4)	
C15	0.4160 (2)	0.85547 (14)	0.30742 (12)	0.0242 (4)	
C16	0.1122 (3)	0.83399 (18)	0.29660 (16)	0.0381 (6)	
H16A	0.0946	0.8713	0.3425	0.057*	
H16B	0.1330	0.7679	0.3133	0.057*	
H16C	0.0164	0.8373	0.2521	0.057*	
C17	0.1126 (3)	1.00169 (16)	0.16858 (16)	0.0369 (5)	
H17A	0.0125	0.9693	0.1711	0.055*	
H17B	0.1151	1.0130	0.1119	0.055*	
H17C	0.1185	1.0623	0.1972	0.055*	
C18	0.4682 (3)	1.03754 (16)	0.15589 (15)	0.0381 (5)	
H18A	0.5041	1.0930	0.1899	0.057*	
H18B	0.3788	1.0554	0.1112	0.057*	
H18C	0.5572	1.0136	0.1340	0.057*	
C19	0.6924 (2)	0.92654 (18)	0.29768 (18)	0.0445 (6)	
H19A	0.7412	0.9294	0.2507	0.067*	
H19B	0.7397	0.8741	0.3329	0.067*	
H19C	0.7122	0.9861	0.3278	0.067*	
C20	0.4730 (3)	0.79563 (19)	0.38119 (14)	0.0437 (6)	
H20A	0.5827	0.7739	0.3832	0.065*	
H20B	0.4020	0.7408	0.3789	0.065*	
H20C	0.4716	0.8330	0.4297	0.065*	
I1	0.651593 (15)	0.699360 (11)	0.219599 (10)	0.03611 (5)	
Ti1	0.37146 (3)	0.80034 (2)	0.169855 (19)	0.01689 (6)	

### Atomic displacement parameters (Å^2^)

**Table:**

	*U*^11^	*U*^22^	*U*^33^	*U*^12^	*U*^13^	*U*^23^
C1	0.0265 (8)	0.0231 (9)	0.0196 (9)	−0.0047 (8)	0.0047 (7)	−0.0004 (9)
C2	0.0195 (7)	0.0234 (10)	0.0167 (9)	−0.0002 (7)	0.0032 (6)	−0.0020 (7)
C3	0.0237 (8)	0.0228 (10)	0.0146 (8)	−0.0018 (7)	0.0023 (7)	0.0009 (7)
C4	0.0244 (9)	0.0291 (11)	0.0195 (10)	0.0003 (7)	0.0077 (7)	−0.0042 (8)
C5	0.0296 (9)	0.0219 (10)	0.0235 (10)	0.0026 (7)	0.0039 (8)	−0.0041 (8)
C6	0.0409 (12)	0.0350 (13)	0.0372 (14)	−0.0143 (10)	0.0098 (10)	0.0050 (10)
C7	0.0192 (8)	0.0413 (13)	0.0307 (12)	0.0032 (8)	0.0018 (8)	−0.0043 (9)
C8	0.0388 (11)	0.0311 (11)	0.0209 (11)	−0.0015 (9)	0.0031 (8)	0.0079 (9)
C9	0.0353 (11)	0.0577 (16)	0.0329 (13)	0.0027 (11)	0.0195 (10)	−0.0042 (12)
C10	0.0495 (13)	0.0252 (12)	0.0514 (16)	0.0078 (10)	0.0048 (12)	−0.0082 (11)
C11	0.0245 (8)	0.0241 (10)	0.0196 (10)	−0.0020 (7)	0.0096 (7)	−0.0052 (8)
C12	0.0240 (8)	0.0210 (9)	0.0192 (10)	0.0044 (7)	0.0029 (7)	−0.0038 (8)
C13	0.0298 (9)	0.0175 (9)	0.0211 (10)	−0.0025 (7)	0.0086 (8)	−0.0017 (8)
C14	0.0215 (8)	0.0242 (10)	0.0269 (11)	−0.0005 (7)	0.0030 (7)	−0.0053 (8)
C15	0.0324 (10)	0.0217 (10)	0.0169 (10)	0.0020 (8)	0.0022 (8)	0.0003 (8)
C16	0.0411 (12)	0.0407 (13)	0.0405 (14)	−0.0130 (10)	0.0260 (11)	−0.0125 (11)
C17	0.0380 (11)	0.0302 (12)	0.0378 (14)	0.0153 (10)	−0.0013 (10)	−0.0073 (10)
C18	0.0557 (14)	0.0270 (12)	0.0348 (13)	−0.0109 (10)	0.0173 (11)	0.0016 (10)
C19	0.0242 (10)	0.0397 (14)	0.0631 (18)	−0.0047 (9)	−0.0033 (10)	−0.0152 (13)
C20	0.0665 (15)	0.0384 (13)	0.0216 (11)	0.0077 (12)	0.0008 (10)	0.0085 (11)
I1	0.02397 (6)	0.03285 (8)	0.04797 (10)	0.01242 (6)	0.00080 (5)	−0.00121 (8)
Ti1	0.01598 (12)	0.01732 (15)	0.01722 (15)	0.00272 (12)	0.00345 (11)	0.00109 (14)

### Geometric parameters (Å, °)

**Table:**

C1—C2	1.411 (3)	C11—C12	1.414 (3)
C1—C5	1.416 (3)	C11—C16	1.501 (3)
C1—C6	1.501 (3)	C11—Ti1	2.3760 (18)
C1—Ti1	2.3864 (19)	C12—C13	1.417 (3)
C2—C3	1.422 (2)	C12—C17	1.501 (3)
C2—C7	1.504 (2)	C12—Ti1	2.3862 (19)
C2—Ti1	2.3869 (17)	C13—C14	1.409 (3)
C3—C4	1.413 (3)	C13—C18	1.497 (3)
C3—C8	1.503 (3)	C13—Ti1	2.3726 (19)
C3—Ti1	2.3729 (19)	C14—C15	1.409 (3)
C4—C5	1.414 (3)	C14—C19	1.509 (3)
C4—C9	1.500 (3)	C14—Ti1	2.416 (2)
C4—Ti1	2.396 (2)	C15—C20	1.497 (3)
C5—C10	1.506 (3)	C15—Ti1	2.406 (2)
C5—Ti1	2.422 (2)	C16—H16A	0.9800
C6—H6A	0.9800	C16—H16B	0.9800
C6—H6B	0.9800	C16—H16C	0.9800
C6—H6C	0.9800	C17—H17A	0.9800
C7—H7A	0.9800	C17—H17B	0.9800
C7—H7B	0.9800	C17—H17C	0.9800
C7—H7C	0.9800	C18—H18A	0.9800
C8—H8A	0.9800	C18—H18B	0.9800
C8—H8B	0.9800	C18—H18C	0.9800
C8—H8C	0.9800	C19—H19A	0.9800
C9—H9A	0.9800	C19—H19B	0.9800
C9—H9B	0.9800	C19—H19C	0.9800
C9—H9C	0.9800	C20—H20A	0.9800
C10—H10A	0.9800	C20—H20B	0.9800
C10—H10B	0.9800	C20—H20C	0.9800
C10—H10C	0.9800	I1—Ti1	2.7508 (3)
C11—C15	1.413 (3)		
			
C2—C1—C5	107.64 (16)	C11—C15—Ti1	71.66 (11)
C2—C1—C6	125.89 (18)	C20—C15—Ti1	125.22 (15)
C5—C1—C6	125.6 (2)	C11—C16—H16A	109.5
C2—C1—Ti1	72.83 (11)	C11—C16—H16B	109.5
C5—C1—Ti1	74.25 (11)	H16A—C16—H16B	109.5
C6—C1—Ti1	126.88 (14)	C11—C16—H16C	109.5
C1—C2—C3	108.31 (15)	H16A—C16—H16C	109.5
C1—C2—C7	122.90 (17)	H16B—C16—H16C	109.5
C3—C2—C7	126.92 (18)	C12—C17—H17A	109.5
C1—C2—Ti1	72.79 (10)	C12—C17—H17B	109.5
C3—C2—Ti1	72.08 (10)	H17A—C17—H17B	109.5
C7—C2—Ti1	133.24 (13)	C12—C17—H17C	109.5
C4—C3—C2	107.64 (17)	H17A—C17—H17C	109.5
C4—C3—C8	124.22 (18)	H17B—C17—H17C	109.5
C2—C3—C8	127.44 (17)	C13—C18—H18A	109.5
C4—C3—Ti1	73.64 (11)	C13—C18—H18B	109.5
C2—C3—Ti1	73.16 (11)	H18A—C18—H18B	109.5
C8—C3—Ti1	126.33 (13)	C13—C18—H18C	109.5
C3—C4—C5	108.02 (17)	H18A—C18—H18C	109.5
C3—C4—C9	124.64 (19)	H18B—C18—H18C	109.5
C5—C4—C9	127.19 (19)	C14—C19—H19A	109.5
C3—C4—Ti1	71.88 (11)	C14—C19—H19B	109.5
C5—C4—Ti1	73.95 (12)	H19A—C19—H19B	109.5
C9—C4—Ti1	123.42 (14)	C14—C19—H19C	109.5
C4—C5—C1	108.31 (17)	H19A—C19—H19C	109.5
C4—C5—C10	126.7 (2)	H19B—C19—H19C	109.5
C1—C5—C10	124.3 (2)	C15—C20—H20A	109.5
C4—C5—Ti1	71.92 (11)	C15—C20—H20B	109.5
C1—C5—Ti1	71.50 (11)	H20A—C20—H20B	109.5
C10—C5—Ti1	130.03 (16)	C15—C20—H20C	109.5
C1—C6—H6A	109.5	H20A—C20—H20C	109.5
C1—C6—H6B	109.5	H20B—C20—H20C	109.5
H6A—C6—H6B	109.5	C13—Ti1—C3	98.28 (7)
C1—C6—H6C	109.5	C13—Ti1—C11	57.88 (6)
H6A—C6—H6C	109.5	C3—Ti1—C11	117.72 (7)
H6B—C6—H6C	109.5	C13—Ti1—C12	34.66 (6)
C2—C7—H7A	109.5	C3—Ti1—C12	91.73 (7)
C2—C7—H7B	109.5	C11—Ti1—C12	34.53 (7)
H7A—C7—H7B	109.5	C13—Ti1—C1	141.80 (7)
C2—C7—H7C	109.5	C3—Ti1—C1	57.69 (6)
H7A—C7—H7C	109.5	C11—Ti1—C1	104.38 (6)
H7B—C7—H7C	109.5	C12—Ti1—C1	110.89 (7)
C3—C8—H8A	109.5	C13—Ti1—C2	108.81 (7)
C3—C8—H8B	109.5	C3—Ti1—C2	34.76 (6)
H8A—C8—H8B	109.5	C11—Ti1—C2	94.60 (6)
C3—C8—H8C	109.5	C12—Ti1—C2	84.08 (6)
H8A—C8—H8C	109.5	C1—Ti1—C2	34.38 (6)
H8B—C8—H8C	109.5	C13—Ti1—C4	120.53 (7)
C4—C9—H9A	109.5	C3—Ti1—C4	34.48 (7)
C4—C9—H9B	109.5	C11—Ti1—C4	150.89 (7)
H9A—C9—H9B	109.5	C12—Ti1—C4	125.37 (7)
C4—C9—H9C	109.5	C1—Ti1—C4	57.33 (7)
H9A—C9—H9C	109.5	C2—Ti1—C4	57.18 (6)
H9B—C9—H9C	109.5	C13—Ti1—C15	57.21 (7)
C5—C10—H10A	109.5	C3—Ti1—C15	148.49 (7)
C5—C10—H10B	109.5	C11—Ti1—C15	34.37 (6)
H10A—C10—H10B	109.5	C12—Ti1—C15	56.77 (7)
C5—C10—H10C	109.5	C1—Ti1—C15	128.41 (7)
H10A—C10—H10C	109.5	C2—Ti1—C15	128.66 (6)
H10B—C10—H10C	109.5	C4—Ti1—C15	173.78 (7)
C15—C11—C12	107.42 (16)	C13—Ti1—C14	34.20 (7)
C15—C11—C16	124.58 (19)	C3—Ti1—C14	131.07 (7)
C12—C11—C16	127.57 (19)	C11—Ti1—C14	56.95 (6)
C15—C11—Ti1	73.97 (11)	C12—Ti1—C14	56.55 (6)
C12—C11—Ti1	73.13 (11)	C1—Ti1—C14	161.04 (7)
C16—C11—Ti1	124.40 (14)	C2—Ti1—C14	140.24 (6)
C11—C12—C13	108.52 (16)	C4—Ti1—C14	140.74 (7)
C11—C12—C17	125.67 (18)	C15—Ti1—C14	33.98 (7)
C13—C12—C17	123.65 (19)	C13—Ti1—C5	153.93 (7)
C11—C12—Ti1	72.34 (11)	C3—Ti1—C5	56.98 (7)
C13—C12—Ti1	72.15 (11)	C11—Ti1—C5	137.70 (7)
C17—C12—Ti1	134.62 (14)	C12—Ti1—C5	140.73 (7)
C14—C13—C12	107.21 (17)	C1—Ti1—C5	34.24 (6)
C14—C13—C18	125.77 (18)	C2—Ti1—C5	56.64 (6)
C12—C13—C18	126.13 (19)	C4—Ti1—C5	34.13 (7)
C14—C13—Ti1	74.60 (11)	C15—Ti1—C5	148.74 (7)
C12—C13—Ti1	73.20 (11)	C14—Ti1—C5	162.29 (6)
C18—C13—Ti1	126.25 (15)	C13—Ti1—I1	110.43 (5)
C13—C14—C15	108.57 (16)	C3—Ti1—I1	123.17 (5)
C13—C14—C19	124.2 (2)	C11—Ti1—I1	119.10 (5)
C15—C14—C19	126.6 (2)	C12—Ti1—I1	138.03 (5)
C13—C14—Ti1	71.20 (11)	C1—Ti1—I1	107.72 (5)
C15—C14—Ti1	72.60 (11)	C2—Ti1—I1	137.84 (5)
C19—C14—Ti1	128.94 (15)	C4—Ti1—I1	89.38 (5)
C14—C15—C11	108.14 (17)	C15—Ti1—I1	86.35 (5)
C14—C15—C20	125.85 (19)	C14—Ti1—I1	81.82 (5)
C11—C15—C20	125.8 (2)	C5—Ti1—I1	81.21 (5)
C14—C15—Ti1	73.42 (12)		
